# Identification and Characterization of *EI* (*Elongated Internode*) Gene in Tomato (*Solanum lycopersicum*)

**DOI:** 10.3390/ijms20092204

**Published:** 2019-05-05

**Authors:** Xiaorong Sun, Jinshuai Shu, Ali Mohamed Ali Mohamed, Xuebin Deng, Xiaona Zhi, Jinrui Bai, Yanan Cui, Xiaoxiao Lu, Yongchen Du, Xiaoxuan Wang, Zejun Huang, Yanmei Guo, Lei Liu, Junming Li

**Affiliations:** Institute of Vegetables and Flowers, Chinese Academy of Agricultural Sciences, Key Laboratory of Biology and Genetic Improvement of Horticultural Crops, Ministry of Agriculture, 12 Zhongguancun Nandajie Street, Beijing 100081, China; sunxiaorong@caas.cn (X.S.); shujinshuai@caas.cn (J.S.); almuttaried@hotmail.com (A.M.A.M.); dengxuebin@caas.cn (X.D.); 18763825752@163.com (X.Z.); baijinrui@caas.cn (J.B.); 15621567802@163.com (Y.C.); luxiaoxiao914@163.com (X.L.); duyongchen@caas.cn (Y.D.); wangxiaoxuan@caas.cn (X.W.); huangzejun@caas.cn (Z.H.); guoyanmei@caas.cn (Y.G.)

**Keywords:** tomato, *Elongated Internode* (*EI*), QTL, GA2ox7

## Abstract

Internode length is an important agronomic trait affecting plant architecture and crop yield. However, few genes for internode elongation have been identified in tomato. In this study, we characterized an elongated internode inbred line P502, which is a natural mutant of the tomato cultivar 05T606. The mutant P502 exhibits longer internode and higher bioactive GA concentration compared with wild-type 05T606. Genetic analysis suggested that the elongated internode trait is controlled by quantitative trait loci (QTL). Then, we identified a major QTL on chromosome 2 based on molecular markers and bulked segregant analysis (BSA). The locus was designated as *EI* (*Elongated Internode*), which explained 73.6% genetic variance. The *EI* was further mapped to a 75.8-kb region containing 10 genes in the reference Heinz 1706 genome. One single nucleotide polymorphism (SNP) in the coding region of *solyc02g080120.1* was identified, which encodes gibberellin 2-beta-dioxygenase 7 (SlGA2ox7). SlGA2ox7, orthologous to AtGA2ox7 and AtGA2ox8, is involved in the regulation of GA degradation. Overexpression of the wild *EI* gene in mutant P502 caused a dwarf phenotype with a shortened internode. The difference of *EI* expression levels was not significant in the P502 and wild-type, but the expression levels of GA biosynthetic genes including *CPS*, *KO*, *KAO*, *GA20ox1*, *GA20ox2*, *GA20ox4*, *GA3ox1*, *GA2ox1*, *GA2ox2*, *GA2ox4*, and *GA2ox5*, were upregulated in mutant P502. Our results may provide a better understanding of the genetics underlying the internode elongation and valuable information to improve plant architecture of the tomato.

## 1. Introduction

Plant height is an important component of plant architecture, and is highly correlated with the yield [1]. One of the decisive factors affecting plant height is internode length. The reduced plant height or internode length of semi-dwarf varieties has improved the harvest index and biomass production. The introduction of the “green revolution” semi-dwarf gene *SD1* in rice and *Rht* in wheat resulted in substantial increases in grain yields and helped to avert predicted food shortages in Asia during the 1960s and 1970s [2,3,4,5]. To explore the genetic potential, several genes or quantitative trait loci (QTL) controlling the internode length in rice [6,7,8], maize [9,10,11], wheat [12,13], and sorghum [14,15] have been identified. In a recent report, the semi-dwarf gene *SBI* was cloned, which could shorten the basal internode of rice. Moreover, the *SBI* allele-introduced varieties have great potential for improving lodging resistance and yield [16].

The molecular genetic analysis of dwarf and slender mutants revealed that most of the mutations are related to the biosynthesis pathways and signal transduction of phytohormones, mostly gibberellins (GAs) [2,9,17,18,19]. GAs are a group of tetracyclic diterpenoid that affect plant developmental processes such as stem elongation [20]. Nowadays, more than 130 GAs have been identified, but relatively few GAs (e.g., GA_1_, GA_3_, GA_4_, and GA_7_) have intrinsic biological activity [21]. The rice *SD1* encodes GA20ox2 and catalyzes the conversion of GA_12_/GA_53_ to bioactive GA precursors GA_9_/GA_20_. The recessive semi-dwarf mutant *sd1* can be restored by exogenous GA_3_ [22,23]. DELLA protein is a negative regulatory factor in the GA signaling pathway, which inhibits plant growth. The semi-dominant mutations that occurred in Arabidopsis *GAI*, maize *D8*, wheat *Rht*, and rice *GAI* were caused by the gain of function of DELLA protein, which led to the dwarfism [3,24,25,26,27,28]. In contrast, the loss-of-function of DELLA protein in barley *SLN1*, rice *SLR1*, and tomato *PRO* increased growth and caused a GA-constitutive response phenotype [29,30,31]. In addition, other phytohormones including brassinosteroid (BR), indole-3-acetic acid (IAA), and strigolactones (SLs) have also been proven to be involved in the regulation of internode length or plant height [7,14,32,33,34].

Tomato (*Solanum lycopersicum*) is the second most consumed vegetable crop and is widely grown around the world [35]. The tomato internode length not only affects the plant architecture, but also plays an important role in mechanized harvesting. However, there are few reports on the genetic regulation of internode length in tomato. Evidence has shown that the well-known dwarf cultivar Micro-Tom, which has the characteristics of extreme dwarfing, dark and wrinkled leaves, has at least two mutations (*d* and *mnt*) affecting internode length [36]. However, Micro-Tom was produced for ornamental purposes, and as a conventional model system for research due to its small size, rapid growth, and easy transformation. Due to its characteristics of extreme dwarfing, it seems very limited in a practical breeding program. Moreover, the tomato *br* locus contributes to a shorter internode and could be useful source for tomato short internode breeding. However, the *br* locus was only mapped to a 763.1-kb region on chromosome 1, and the gene has not been cloned yet [37]. Therefore, to clone new gene or locus that controls internode elongation is of great theoretical and practical significance. It is helpful to clarify the regulatory mechanism of tomato internode elongation and to provide the possibility of establishing a breeding approach. Furthermore, with the completion of tomato genome sequencing as well as the rapid development of sequencing, marker development has become easier, which has accelerated the speed of gene cloning [38].

In this study, we performed a phenotypic characterization of the mutant P502, which shows a significantly elongated internode compared with wild-type 05T606. Then, we report on the molecular identification of the *EI* (*Elongated Internode*) gene by map-based cloning. *EI*, which is expressed in the root, stem, and leaf, encodes the GA2ox7 enzyme involved in the GA metabolism pathway. Overexpressing of *EI* can cause a dwarf phenotype with short internode. Our results indicate that *EI* plays an important role in controlling the tomato internode elongation.

## 2. Results

### 2.1. Characterization of Elongated Internode Inbred Line P502

**Figure 1:**
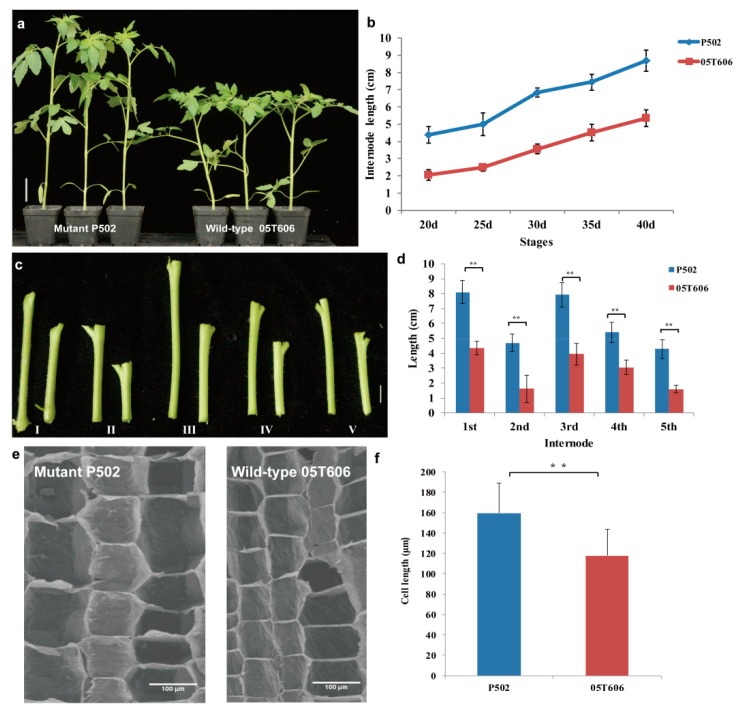
The phenotypic characterization of the mutant P502 and wild-type 05T606 at 40-day-old seedlings. Morphological phenotypes (**a**) and the statistical data of internode lengths (**b**) of the mutant P502 and wild-type 05T606. (**c**) Morphological phenotypes of the first five internode lengths of the mutant P502 (left) and wild-type (right). (**d**) Statistical data of internode length in (**c**). Longitudinal sections (**e**) and the statistical data (**f**) of the third internode pith cell length of P502 and wild-type. Scale bar is 5 cm in (**a**), 1 cm in (**c**), 100 μm in (**e**). A Student’s *t* test indicated a significant difference ((**b**,**d**), *n* = 30 plants; (**f**) *n* = 90 cells) in (**d**,**f**). ** *p* < 0.01. All data are given as mean ± SD.

To compare the dynamic difference in the internode lengths of P502 and wild-type 05T606, we recorded the mean internode lengths of 20-day-old, 25-day-old, 30-day-old, 35-day-old, and 40-day-old seedlings. The results showed that P502 had a longer internode than the wild-type across the seedling stages (Figure 1a,b). Additionally, we compared individual internode lengths including the first, second, third, fourth, and fifth internodes of 40-day-old seedlings. The results indicated that each internode of P502 was significantly greater than the corresponding internode of the wild-type (Figure 1c,d). An analysis of the longitudinal sections of the third internode with a scanning electron microscope revealed that the cells were much longer in P502 than in the wild-type (Figure 1e,f). These results suggest that the mutant P502 phenotype is characterized by the elongated internode.

### 2.2. Elongated Internode Mutation Is Related to the GA Metabolic Pathway

**Figure 2:**
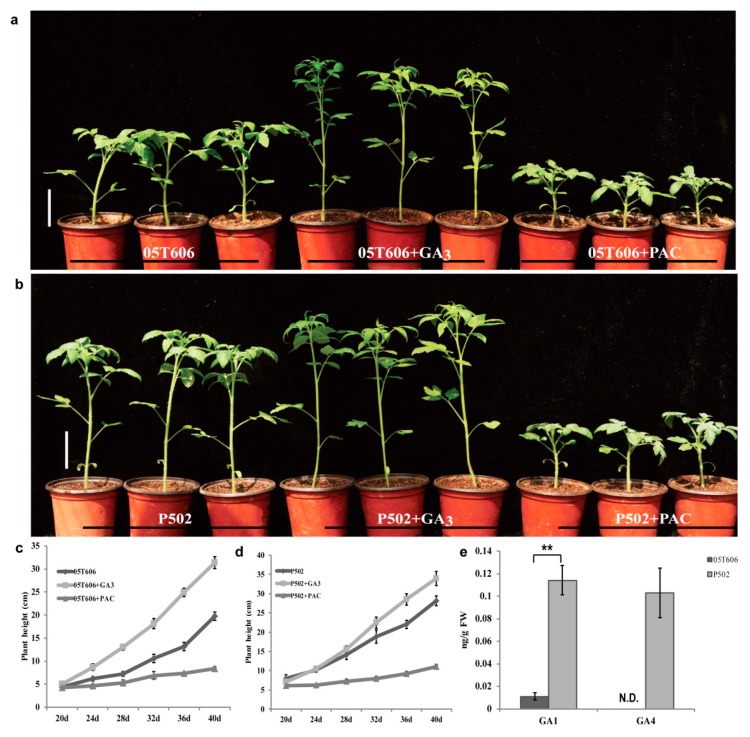
The line P502 is a GA-sensitive mutant. (**a**,**b**) The morphological phenotypes of 30-day-old wild-type and P502 seedlings after treatment with GA_3_ (10^−5^ M) and PAC (10^−7^ M, GA biosynthesis inhibitor), respectively. (**c**,**d**) The statistical data of wild-type and P502 plant height in different stages, respectively. (**e**) Concentration of endogenous bioactive GAs in the first five internodes of 30-day-old mutant P502 and wild-type seedlings. The water treatment was used as control and the scale bar is 5 cm in (**a**,**b**). Data for (**c**,**d**) are based on three replicates of eight plants per group. N.D. represents not detectable. Data for (**e**) are based on three independent biological replicates, and the asterisk indicates a statistically significant difference (Student’s *t*-test, ** *p* < 0.01). All data are given as mean ± SD.

The GAs stimulate cell elongation, and are effective internode elongation regulators. Paclobutrazol (PAC) inhibits the oxidation of *ent*-kaurene, an early step in GA biosynthesis, and can reduce endogenous GA level [39]. To examine the responses to GAs, we sprayed 20-day-old wild-type 05T606 and mutant P502 seedlings with exogenous GA_3_ and PAC. For the 40-day-old seedlings, the plant height of 05T606 and P502 increased by 58.2% and 20.5%, respectively, after GA_3_ treatment. However, the PAC treatment decreased the height of 05T606 by 57.6%, and decreased the height of P502 by 61.0% (Figure 2a–d). Next, we measured the endogenous GA concentration of the first five internodes in 30-day-old seedlings of P502 and 05T606 plants. There was an increase in the bioactive GA_1_ in P502, and bioactive GA_4_ was only detected in mutant P502 (Figure 2e). These results indicate that the elongated internode of mutant P502 is related to the GA metabolic pathway and was caused by a higher level of bioactive GAs.

### 2.3. Genetic Analysis of the Elongated Internode Trait

**Figure 3:**
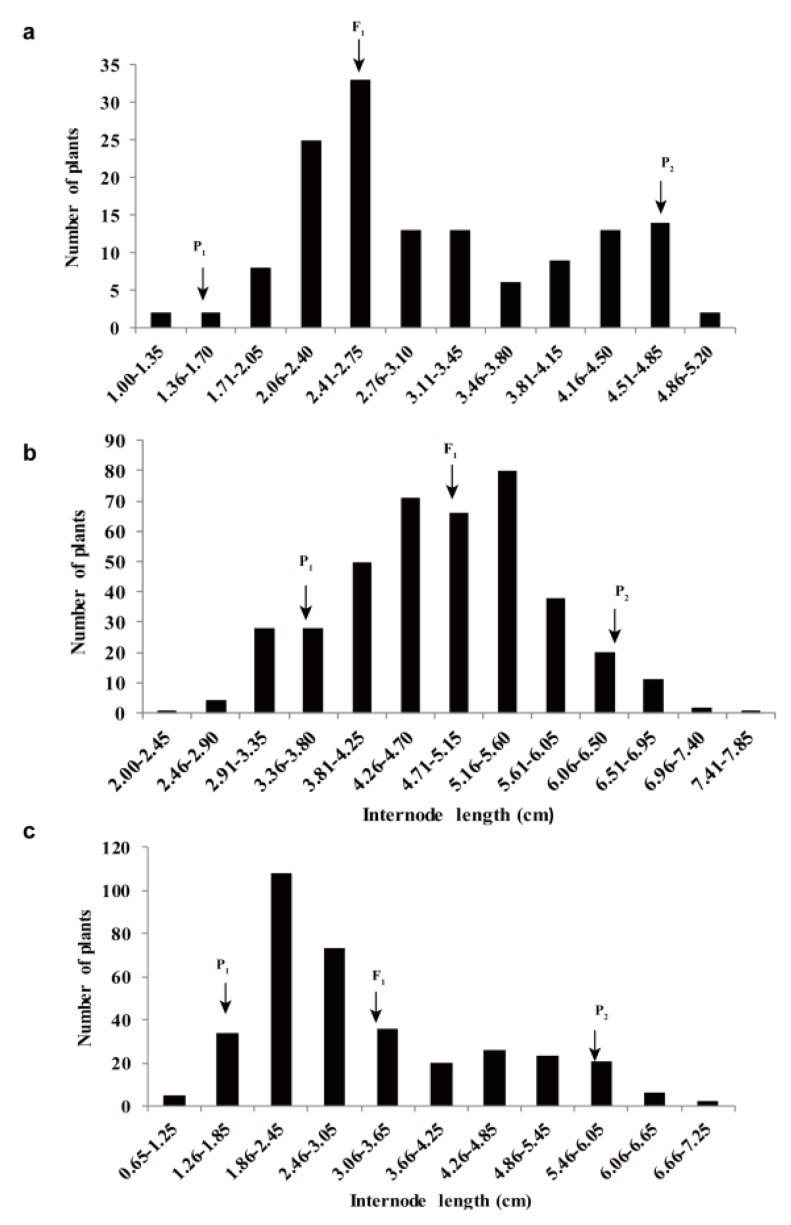
Internode length frequency distribution of F_2_ individuals at 40-day-old seedlings. (**a**) 2016 (*n* = 140). (**b**) 2017 (*n* = 400). (**c**) 2018 (*n* = 354). The mean internode lengths were recorded from the first internode to the fifth internode (starting from the cotyledons).

To determine whether the elongated internode trait is controlled by a single gene or QTL, the first five average internode lengths of the P_1_ (Heinz 1706), P_2_ (P502), F_1_ (Heinz 1706 × P502), and F_2_ population were recorded in the spring of 2016, 2017, and 2018. The internode length frequency of the F_2_ population exhibited a continuous and skewed distribution in different years (Figure 3), and the F_1_ was biased toward the parent Heinz 1706. Moreover, the differences of P_1_, P_2_, and F_1_ were significant (Table 1). These results indicate that the elongated internode length is a quantitative trait and that this population was ideal for elongated internode QTL analysis.

### 2.4. Map-Based Cloning of the EI Gene

A total of 372 InDel markers distributed on 12 chromosomes were screened, and 90 were polymorphic between the parents. Of these 90 markers, four markers (D55, D57, D64, and D67) located on chromosome 2 were polymorphic between the E and N bulks. The bands of the E pool were consistent with those of the parent P502, whereas the bands of the N pool were heterozygous. These results suggest that the locus is present on chromosome 2.

**Figure 4:**
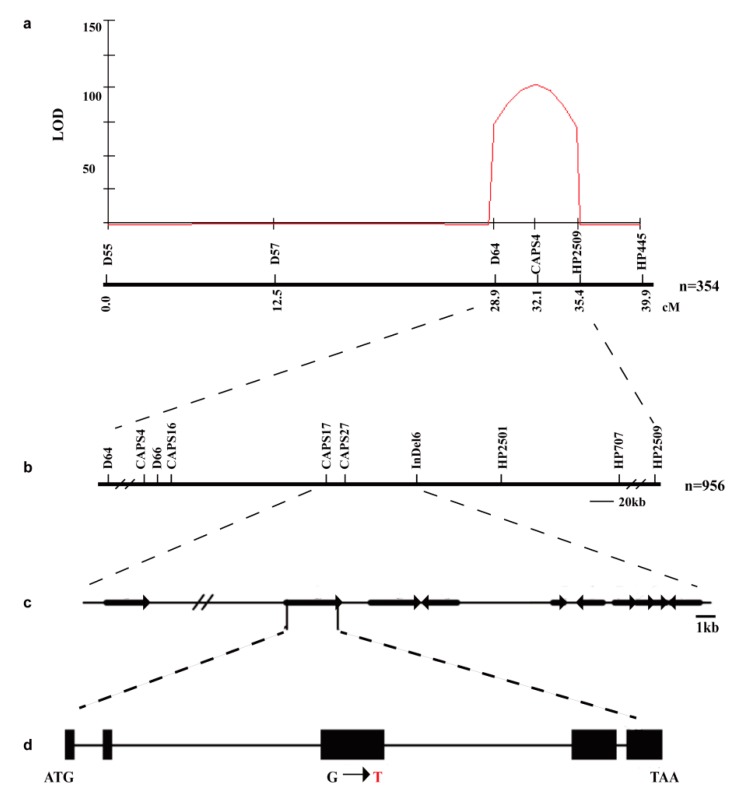
Map-based cloning of *ei* locus. (**a**) The genetic map of *ei* on chromosome 2, mapped using 354 F_2_ individuals and six polymorphic markers. (**b**) The *ei* locus was fine-mapped to the interval between markers CAPS17 and InDel6. (**c**) Predicted genes in the region encompassing the *ei* locus. The arrows indicate the direction of transcription. (**d**) Gene structure and the mutation site. The black rectangles and black line indicate exons and introns, respectively. The red letter represents the mutation base in mutant P502.

The 354 F_2_ individuals, derived from Heinz 1706 × P502, were used for QTL analysis. The results showed that there was only a single peak, with a logarithm of odds score of 102.4 explaining 73.6% phenotypic variance (Figure 4a). Therefore, we concluded that the locus named *elongated internode* (*ei*) was located between markers D64 and HP2509. Another 956 F_2_ individuals were screened for recombinants with the flanking markers D64 and HP2509. The detected recombinants were analyzed with another seven CAPS and InDel markers between the flanking markers (Figure 4b). According to the genotypes of the recombinants and the phenotypes of F_3_ individuals, we narrowed the *ei* locus to an interval between CAPS17 and InDel6 (Table 2), corresponding to a 75.8-kb region on chromosome 2 of the reference Heinz 1706 genome.

The Solanaceae Genomics Network website (SGN; http://solgenomics.net) searches [40] indicated that there were 10 genes in this region (Figure 4c, Table 3). By analyzing the sequenced P502 genome, we determined that the DNA sequence had no mutations in the other nine genes, whereas *solyc02g080120.1* contained a SNP (G–T) in the exon region (Figure 4d). Thus, *solyc02g080120.1* was amplified using genomic DNA extracted from 05T606 and P502 plants and eight primer pairs (A1, A2, A3, A4, A5, A6, A7, and A8; Table S1). The amplification results revealed that the *EI* gene consists of 4626 bp (with five exons and four introns). Moreover, the *EI* gene in mutant P502 includes a SNP mutation in the third exon (G2152T) (Figure S1). To confirm this mutation site, the *solyc02g080120.1* coding sequence (CDS) was amplified by RT-PCR (CDS-F and CDS-R primers) (Table S1). Sequences of the CDS further confirmed the presence of a SNP mutation in the coding region, which resulted in an amino acid mutation in mutant P502.

To determine whether the G-to-T transition was directly associated with the elongated internode phenotype, a co-segregation analysis was conducted with a functional marker (KASP) developed from this SNP. The KASP marker (S-A1: GAAGGTGACCAAGTTCATGCTCACAAGCTTCACAAGAATGGGG; S-A2: GAAGGTCGGAGTCAACGGATTGCACAAGCTTCACAAGAATGGGT; S-C: GTGATACTCCATGGTTTACAACTTGGAA) was used to validate the genotypes of 354 F_2_ individuals derived from the cross of Heinz 1706 × P502 hybridization. The results showed that this KASP marker was co-segregated with internode length (Figure S2), with a 100% accuracy rate. It further confirmed that SNP mutation is associated with the elongated internode.

### 2.5. Protein Sequence Alignment and Phylogenetic Analysis of SlGA2ox7

**Figure 5:**
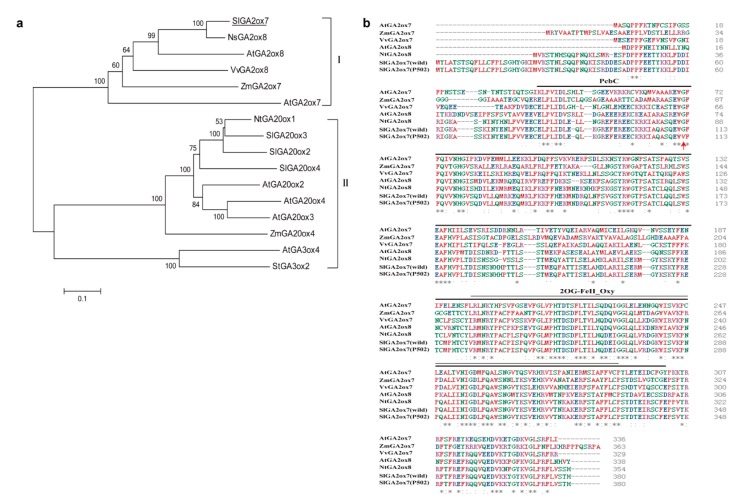
Phylogenetic analysis and sequence alignment of SlGA2ox7 with various species. (**a**) Phylogenetic analysis of SlGA2ox7. The phylogenetic tree was generated using the neighbor-joining method built in MEGA6.0, and the inferred phylogeny was tested by bootstrap analysis with 1000 replicate datasets. Numbers shown at the tree forks indicate the frequency of occurrence among all bootstrap iterations performed. (**b**) Alignment of the SlGA2ox7 sequences. The proteins were aligned with the ClustalW program. The bold black line and the thin black line indicate the PcbC domain (81–343) and 2OG-FeII_Oxy domain (237–332), respectively. The red arrow represents the amino acid at position 112 of SlGA2ox7.

According to the gene annotation, *EI* encodes the SlGA2ox7 enzyme, which comprises 380 amino acids. The sequence alignment and phylogenetic analysis revealed that GA2ox7 and GA2ox8 are clustered in one group, which is separate from GA20oxs and GA3oxs (Figure 5a), indicating that GA2ox7 and GA2ox8 are conserved in tobacco, Arabidopsis, maize, and grape. Moreover, amino acid position 112 of wild-type SlGA2ox7 is a hydrophilic glycine, whereas it is a hydrophobic valine in P502. The SlGA2ox7 at this position is located within a conserved region of the PcbC superfamily (Figure 5b), indicating that the altered hydrophobicity of the amino acid may affect the function of SlGA2ox7.

### 2.6. Overexpression of Wild-Type EI in P502 Resulted in Dwarfism

**Figure 6:**
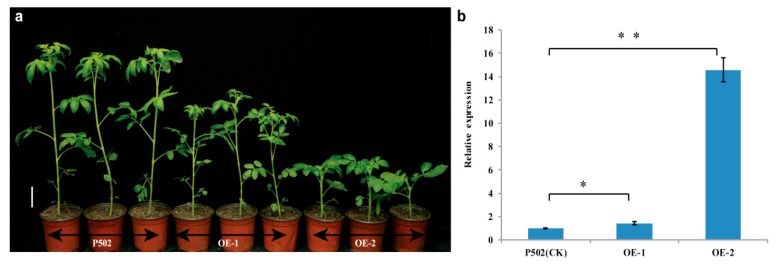
Overexpression of wild-type *EI* gene reduced the internode length of mutant P502. (**a**) Phenotype of P502 (CK) and T_1_ transgenic plants (OE-1 and OE-2) at 40-day-old seedlings. (**b**) Expression level of *EI* in T_1_ lines (OE-1 and OE-2). Total RNA was isolated from internode of P502 and transgenic T_1_ plants at 40-day-old seedlings. Data represent mean ± SD based on three independent biological and three technical experiments. Scale bar is 5 cm in (**a**). Statistical significances were calculated based on two-tailed, two-sample Student’s *t*-test at * *p* < 0.05 and ** *p* < 0.01.

To confirm the function of *EI*, the recombinant plasmid 35S: *EI* was introduced into the mutant P502. Transgenic plants were obtained after screening for regenerated shoots on selection medium containing kanamycin. The transgenic plants were analyzed further by PCR with primers NPTII-F and NPTII-R, and two positive transgenic plants (T_0_-1 and T_0_-2) were obtained. The *EI*-overexpressing transgenic T_1_ homozygous lines (OE-1 and OE-2) exhibited dwarfism with shortened internodes (CK: 36.00 ± 1.32 cm; OE-1: 24.7 ± 1.14 cm; OE-2: 8.53 ± 0.91 cm) (Figure 6a). The *EI* expression level was 1.44-fold and 14-fold higher in the OE-1 and OE-2 plants, respectively, than in the P502 control plants (Figure 6b). These results indicated that overexpression of *EI* could result in dwarf phenotype with shortened internodes, and the degree of shortness is related to the expression level of *EI*.

### 2.7. The Expression Analysis of GA Metabolic Pathway-Related Genes

**Figure 7:**
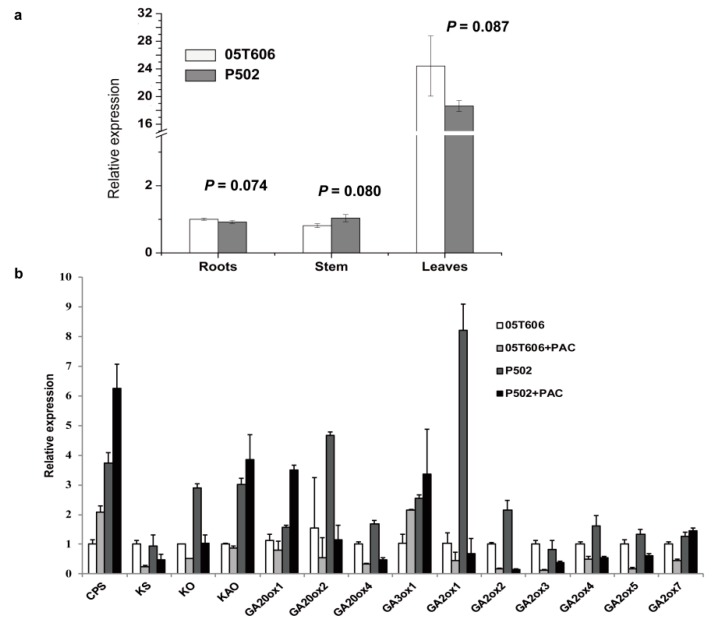
Expression analysis in the qRT-PCR assay. (**a**) Expression patterns of *EI* in the roots, stem, and leaves of P502 and the wild-type. Total RNA was isolated from P502 and wild-type at 40-day-old seedlings. A Student’s *t* test was used for statistical analysis. (**b**) Expression levels of the GA biosynthetic genes after 10^−7^ M paclobutrazol (PAC, GA biosynthesis inhibitor) treatment. Data represent mean ± SD based on three independent biological and three technical experiments.

To study the spatiotemporal expression patterns of *EI*, total RNA was extracted from the leaves, stem (the third internode), and roots of 40-day-old wild-type and mutant P502 seedlings. The results of a qRT-PCR assay indicated that *EI* was expressed in the leaves, stem, and roots (Figure 7a), and the expression level in the leaves was more than 18-fold higher than that in the stem and roots. Interestingly, the differences of expression levels were not significant (*p* > 0.05) in the wild-type and mutant P502 (Figure 7a), indicating the G-to-T mutation does not alter the expression level of the *EI*.

Many genes are involved in the GA biosynthetic pathway. CPS, KS, and KO are each encoded by a single gene in most plant species examined. However, the cytosol-localized GA20ox, GA3ox, and GA2ox each is encoded by a small gene family [41]. The qRT-PCR assay indicated that *CPS*, *KO*, *KAO*, *GA20ox1*, *GA20ox2*, *GA20ox4*, *GA3ox1*, *GA2ox1*, *GA2ox2*, *GA2ox4*, and *GA2ox5*, were more highly expressed in P502 than in the wild-type 05T606. PAC treatment decreased the expression levels of *KS*, *KO*, *GA20ox2*, *GA20ox4*, *GA2ox1*, *GA2ox2*, *GA2ox3*, *GA2ox4*, and *GA2ox5*, but increased the expression level of *CPS* and *GA3ox1*. However, the expression of *EI* (*GA2ox7*) in P502 was not significantly changed after PAC treatment (Figure 7b).

## 3. Discussion

The GA-related mutants have been categorized into GA-deficient (GA-sensitive) mutants and GA-insensitive mutants according to their response to exogenous GAs [42]. In GA-deficient dwarfs, the normal phenotype can be restored by the application of exogenous GAs and the mutations are usually due to a deficiency in the GA metabolic pathway [43]. In GA-insensitive types, the mutants are deficient in GA signaling and exhibit altered GA responses or the constitutive activation of GA responses [29,30,31,44]. In our study, the plant height of wild-type and the mutant P502 increased by 58.2% and 20.5%, respectively, after GA_3_ treatment, whereas PAC treatment decreased the height of the wild-type by 57.6%, and the mutant P502 by 61.0% (Figure 2a–d). These results indicated that the mutant P502 was responsive to GA_3_ and PAC, and the mutation was related to the GA metabolic pathway. In addition, it was found that mutant P502 was far less sensitive to GA_3_ and more sensitive to PAC compared with the wild-type. This may be caused by a higher GA concentration of mutant P502 (Figure 2e), which reduced its sensitivity to GA_3_ and increased its sensitivity to PAC.

GA2ox members are thought to disable GA functions by hydroxylating the C-2 position of active GAs or their precursors. The genes encoding 2-oxidases have been isolated from Arabidopsis, rice, spinach, and pea [45,46,47,48]. However, few GA2-oxidase genes have been isolated from tomato. In our study, we isolated the tomato *EI* gene by map-based cloning. The *EI* gene encodes SlGA2ox7, which belongs to GA2-oxidase. A point mutation in the exon region of *EI* gene resulted in the amino acid mutation (glycine to valine) of SlGA2ox7. GA2ox7 or GA2ox8 is conserved in various species (Figure 5a), and amino acid mutation occurs in the conserved domain of the PcbC superfamily (Figure 5b). Overexpression of *EI* inhibited the internode elongation of mutant P502, leading to dwarfism with a shortened internode (Figure 6a). These results are consistent with the research reported by Schomburg et al. [45], who revealed that the overexpression of *AtGA2ox7* and *AtGA2ox8* induced a dwarf phenotype of Arabidopsis and tobacco. Similar results have been obtained in transgenic plants overexpressing GA 2-oxidases from rice (*O. sativa*) [49]. Taken together, the elongated internode is caused by the loss-of-function of SlGA2ox7.

So far, three different kinds of GA deactivation have been identified. One type of GA2oxs hydroxylates the C-2 of active C_19_-GAs (GA_1_ and GA_4_) or C_19_-GA precursors (GA_20_ and GA_9_) to produce biologically inactive GAs (GA_8_, GA_34_, GA_29_, and GA_51_, respectively) [18]. Another type of GA2oxs including AtGA2ox7, AtGA2ox8, OsGA2ox5, OsGA2ox6, and SoGA2ox3 (*Spinacia oleracea*) accept C_20_-GAs (GA_12_ and GA_53_) as their substrates to produce GA_110_ and GA_97_, respectively [45,50]. In addition, the recombinant SoGA2ox1 can work on both C_19_-GA and C_20_-GA substrates [47]. Although SlGA2ox7 is orthologous to NsGA2ox8 and AtGA2ox8, whether they catalyze the same substrate needs further research.

Bioactive GA (GA_1_, GA_3_, GA_4_, and GA_7_) concentrations are maintained mainly by the balanced activities of GA 3-oxidases (GA3oxs) and GA 20-oxidases (GA20oxs), essential enzymes regulating GA biosynthesis, and GA 2-oxidases (GA2oxs) necessary for GA inactivation [51]. In our study, the expression of *EI* (*GA2ox7*) did not significantly change in the mutant P502 compared with the wild-type 05T606 (Figure 7a), indicating that the mutation of *EI* did not affect its transcript level. However, the expression of GA biosynthesis pathway genes including *CPS*, *KO*, *KAO*, *GA20ox1*, *GA20ox2*, *GA20ox4*, *GA3ox1*, *GA2ox1*, *GA2ox2*, *GA2ox4* and *GA2ox5* were increased (Figure 7b). Similarly, the *dw* mutant of the soybean had lower expression levels of *CPS* and *GA20oxs* than the wild-type [19]. These results indicated that the mutations of genes related to GA synthesis may regulate the expression of other genes involved in the GA biosynthesis pathway by changing the GA concentrations. After exogenous PAC treatment, the growths of the mutant P502 and wild-type 05T606 were blocked. The expression levels of *KS*, *KO*, *GA20ox2*, *GA20ox4*, *GA2ox1*, *GA2ox2*, *GA2ox3*, *GA2ox4*, and *GA2ox5*, were downregulated (Figure 7b), revealing that these genes may be involved in the homeostatic maintenance of bioactive GA levels. Interestingly, the expression of the *EI* (*GA2ox7*) gene in the mutant P502 was not significantly changed after PAC treatment. We speculated that the mutation of *EI* may result in more complex regulation of GA homeostasis.

Tomato cultivars with determinate growth habit, compact, and short internode have been developed for commercial use [35]. The *sp* gene controlling determinate growth habit was cloned and the introduction of the *sp* allele into tomato cultivars has transformed the industry by creating a major modification in plant architecture [52]. However, few genes controlling internode elongation have been cloned. In our study, the *EI* gene for internode elongation was identified by map-based cloning and this gene encodes SlGA2ox7. Increased expression of *EI* caused different degrees of dwarfism, which may provide a useful resource for improving the plant architecture in tomato. Meanwhile, the co-segregated KASP marker developed in our study might be useful for high throughput maker assistant selection (MAS) in short internode breeding programs.

## 4. Materials and Methods

### 4.1. Plant Materials

Three determinate tomato inbred lines, P502, 05T606, and Heinz 1706, were used in this study. The mutant P502 shows an elongated internode, derived from a natural mutant of tomato cultivar 05T606. These were generated at the Institute of Vegetables and Flowers, Chinese Academy of Agricultural Sciences. Heinz 1706 was obtained from the Tomato Genetics Resource Center and displays a normal internode length, which is significantly shorter than the mutant P502 [53].

The mutant P502 (as the male parent) and Heinz 1706 (as the female parent) were hybridized to obtain an F_1_ generation, and F_1_ plants were self-pollinated to generate the F_2_ population, which were used for inheritance analysis and fine mapping. All of the plant materials were grown in a greenhouse in Beijing, China. The average day and night temperatures were set at 25 °C and 20 °C, respectively.

### 4.2. Scanning Electron Microscopy Observation

To measure the cell lengths, the third internode was collected from 40-day-old 05T606 and P502 seedlings. The internode was cut into 5-mm segments and fixed in 3.5% glutaraldehyde for 24 h at room temperature. After washing in 0.1 M phosphate buffer (pH 7.4), the samples were fixed in 1% osmic acid for 2 h, dehydrated in a graded ethanol series, and dried in a Leica-EM CPD 300 desiccator (Leica, Frankfurt, Germany). Longitudinal sections were prepared by cutting the middle of the internode, which was then coated with a gold film. The pith cells at approximately the center of the stem were visualized and photographed with the Hitachi SU 8010 scanning election microscope (Hitachi, Tokyo, Japan). The cell length was measured with IMAGEJ software [54].

### 4.3. Exogenous GA_3_ Treatments and Endogenous GA Quantification

To assess the response of P502 and 05T606 to GAs, the aerial parts of the 20-day-old seedlings were separately sprayed with 10^−5^ M GA_3_ (Sigma, St. Louis Missouri, USA) and 10^−7^ M paclobutrazol (PAC, GA biosynthesis inhibitor; Biotopped, Beijing, China) [55] solutions containing 0.02% Tween-20 at an interval of one day. Control plants were sprayed with water. We sprayed ten times, and stopped at 40-day-old seedlings. The effects of GA_3_ and PAC on stem expansion (from the cotyledons to the uppermost internode) were evaluated every four days. Each treatment was completed with three replicates (each with eight plants).

To determine the concentration of endogenous GAs, the first five internodes from the 30-day-old seedlings of P502 and wild-type 05T606 were collected into three biological replicates. Each biological replicate contained 1 g of tissue fresh weight. Tissue was immediately frozen in liquid nitrogen, and then was stored at −80 °C. The phytohormone extraction and quantitative profiling of GAs (GA_1_, GA_4_, GA_9_, GA_19_, and GA_20_) were performed by HPLC-MS/MS [56].

### 4.4. DNA Extraction and Molecular Marker Development

A single young leaf was collected from each plant at 2-week-old seedlings. Genomic DNA was extracted according to a modified CTAB method [57] and then diluted to a concentration of 100–150 ng/µL in RNase (10 mg/mL) H_2_O (1:100). To develop new markers, the elongated internode line P502 was sequenced with the Illumina HiSeq PE150 system, with a 50× genome coverage (Sequence Read Archive accession number: PRJNA540748). According to differences with the reference genome sequence (Heinz 1706), insertion and deletion (InDel) and competitive allele specific PCR (KASP) markers were designed using Primer Premier 5.0 software [58], and cleaved amplified polymorphic sequence (CAPS) markers were designed by dCAPS Finder 2.0 [59].

### 4.5. Mapping Strategy

The mean internode lengths from the first internode to the fifth internode (starting from the cotyledons) of 40-day-old seedlings were recorded for phenotypic analysis [36,53]. The bulked segregant analysis (BSA) strategy was used for the quick identification of molecular markers linked with the target locus [60]. Two DNA bulks, the elongated internode bulk (E bulk) and the normal internode bulk (N bulk), were generated by pooling equal amounts of DNA from ten elongated internode and ten normal F_2_ plants, respectively.

To screen for polymorphic makers, the two parents were genotyped with 372 InDel markers across 12 tomato chromosomes (unpublished). All polymorphic markers were used to analyze the two bulked DNA samples. The target chromosome was identified based on the BSA results. Subsequently, QTL mapping was conducted according to the internode lengths of 354 F_2_ individuals and genotypes of ideal markers on the target chromosome by using JoinMap 4.0 and MapQTL 6.0 [61,62]. After flanking markers were identified, another 956 F_2_ individuals derived from the same cross were used for selecting recombinants. The F_3_ individuals from eight F_2_ recombinants were obtained for the confirmation of the progeny phenotype. Each F_2:3_ contained 60 plants, and the internode lengths were evaluated on 40-day-old seedlings. Details regarding the polymorphic markers are provided in Table 4.

### 4.6. RNA Extraction and qRT-PCR

Total RNA was extracted using an RNA pure kit (Aidlab, Beijing, China) following the user manual. First-strand cDNA was synthesized using TransScript One-Step gDNA Removal and cDNA Synthesis SuperMix (Transgene, Beijing, China). A quantitative real-time polymerase chain reaction (qRT-PCR) assay was conducted using a SYBR Green reagent (Yeasen, Shanghai, China) and the LightCycler 480 Real-Time detection system (Roche, Basel, Switzerland). Housekeeping gene *actin* (*solyc03g078400*) was used as an internal control to normalize the data. Details regarding the qRT-PCR primers are listed in Table S1 [63]. The qRT-PCR data for each sample were validated with three biological and three technical replicates. The relative expression levels were quantified according to the 2^−ΔΔCt^ method [64].

### 4.7. Protein Sequence Alignment and Phylogenetic Analysis

The sequence was retrieved from the National Center for Biotechnology Information database (NCBI). The BLASTP program [65] was used for homology searches in GenBank. A multiple protein sequence alignment was performed by the ClustalW program, and the phylogenetic tree was constructed according to the neighbor-joining method of the MEGA 6.0 program with 1000 bootstrap replicates [66].

### 4.8. Plasmid Construction and Transformation

The genomic clone including the whole *EI* coding region was amplified from full-length cDNA of wild-type 05T606 with primers OE-F (5′-CACGGGGGACTCTAGAATGTACTTAGCCACCTCCA-3′) and OE-R (5′-GATCGGGGAAATTCGAGCTCTTAGTGAGTTGAGACAAGAAAC-3′). The amplified fragment was cloned into the *Xba*I and *Sac*I sites of the binary vector *pBI*121 by using an In-Fusion HD Cloning Kit (Takara, Dalian, China) to generate an *EI* transformation plasmid under the control of the CaMV35S promoter. The plasmid mediated by *Agrobacterium tumefaciens* strain GV3101 was transformed into the mutant P502 as described by the method of Sharma et al. [67]. After screening for regenerated shoots on the selection medium, the transgenic plants were further confirmed by PCR using NPTII-F (5′-GACAATCGGCTGCTCTGA-3′) and NPTII-R (5′-AACTCCAGCATGAGATCC-3′) primers. The positive transgenic plants were selected and the T_1_ generation was obtained for phenotypic observation and gene expression analysis.

Accession numbers: SlGA2ox7 (XP_004232746), NsGA2ox8 (NP_001289506.1), AtGA2ox8 (NP_193852.2), AtGA2ox7 (NP_175509.1), VvGA2ox8 (NP_001268435.1), ZmGA2ox7 (NP_001148252.2), NtGA20ox1 (NP_001313089.1), SlGA20ox3 (NP_001234579.1), SlGA20ox2 (NP_001234628.2), SlGA20ox4 (NP_001234363.1), AtGA20ox2 (NP_199994.1), AtGA20ox4 (NP_176294.1), AtGA20ox3 (NP_196337.1), ZmGA20ox4 (NP_001308615.1) StGA3ox2 (NP_001275412.1), and AtGA3ox4 (NP_178149.1).

## Figures and Tables

**Table 1:** Internode lengths of P_1_, P_2_, and F_1_ population plants.

Materials	2016 (cm) ^a^	2017 (cm) ^a^	2018 (cm) ^a^
P_1_ (Heinz 1706)	1.52 ± 0.35 a	1.48 ± 0.63 a	1.85 ± 0.30 a
P_2_ (P502)	4.62 ± 0.19 c	4.66 ± 0.60 c	5.54 ± 0.48 c
F_1_ (Heinz 1706 × P502)	2.74 ± 0.28 b	2.53 ± 0.47 b	3.10 ± 0.24 b

^a^ Values followed by different letters (a, b, and c) are significantly different (*p* < 0.01). *n* = 15 plants.

**Table 2:** Genotypes of F_2_ recombinants and phenotypes of F_2:3_ individuals.

Recombinants	Genotype (F_2_) ^a^										Phenotype(F_3_)	
	D64	CAPS4	D66	CAPS16	CAPS17	CAPS27	InDel6	HP2501	HP707	HP2509	N ^b^	E ^b^
6–11	b	h	h	h	h	h	h	h	h	h	-	-
13–5	b	b	b	h	h	h	h	h	h	h	44	15
7–48	b	b	b	b	h	h	h	h	h	h	45	15
7–71	b	b	b	b	h	h	h	h	h	h	43	14
15–543	b	b	b	b	b	h	h	h	h	h	45	14
17–18	b	b	b	b	b	h	h	h	h	h	45	15
15–741	h	h	h	h	h	h	b	b	b	b	46	14
1–2	h	h	h	h	h	h	b	b	b	b	45	15
1–72	h	h	h	h	h	h	h	b	b	b	44	16
6–21	h	h	h	h	h	h	h	h	h	b	-	-

^a^ b in green backgroud: homozygous like P502; h in gray background: heterozygous; ^b^ N: the number of normal internode plants; E: the number of elongated internode plants; -: no data.

**Table 3:** Ten predicted genes in the 75.8-kb fine mapping interval according to the reference genome.

Gene ID ^a^	Position	Functional Annotation
*solyc02g080110.2*	SL2.50ch02: 44414686..44417879 (+)	Unknown Protein (AHRD V1)
*solyc02g080120.1*	SL2.50ch02: 44432042..44436667 (+)	Gibberellin 2-beta-dioxygenase 7
*solyc02g080130.2*	SL2.50ch02: 44439349..44443883 (+)	Chaperone dnaj-like protein
*solyc02g080140.2*	SL2.50ch02: 44444670..44447584 (-)	cysteine-rich PDZ-binding protein
*solyc02g080150.1*	SL2.50ch02: 44458406..44458810 (+)	uncharacterized LOC101262168
*solyc02g080160.2*	SL2.50ch02: 44460578..44462486 (-)	probable xyloglucan endotransglucosylase/hydrolase protein 8
*solyc02g080170.1*	SL2.50ch02: 44471643..44473397 (+)	pentatricopeptide repeat-containing protein At4g21170
*solyc02g080180.1*	SL2.50ch02: 44474353..44475381 (+)	Probable dolichyl-diphosphooligosaccharide—protein glycosyltransferase subunit 3B
*solyc02g080190.2*	SL2.50ch02: 44477570..44478612 (+)	Nuclear transport factor 2
*solyc02g080200.2*	SL2.50ch02: 44478656..44480593 (-)	pectinesterase-like

^a^ Genes were identified based on the tomato model (ITAG release 2.40, SL2.50) in SGN (https://solgenomics.net/) (Access on 21 June 2014).

**Table 4:** Markers used for mapping of *EI* gene.

Primer Name	Forward Primer (5′-3′)	Reverse primer (5′-3′)	Type	Enzyme
D55	AATGACTTACCTACTGGAAAGC	GATTGATCACCCTTTGGATA	InDel	
D57	GAGACATCACTTTGCCTTTC	AAAAGTCTCTCCGCCTATGT	InDel	
D64	TTGTTACCGCTTACTTTGGT	CACAGCTGTTGATTTCTTCA	InDel	
CAPS4	GCATTGCAACCTATTCTCAC	TCTGTAGTTTCCGTCTTCTT	CAPS	*Hae*III
D66	CGTTGTCTAGGTCAATAGCC	AGGTGTTACACTTTCTACGTCT	InDel	
CAPS16	AGAGAAGGAGGATTCGGGTT	ATAGGGGCATTATCAAAAGG	CAPS	*BsrD*I
CAPS17	TAAGTTAGCCATATAAAAC	AAATGACACAGCGAGACA	CAPS	*Mbo*II
CAPS27	GAGAAAATTATTTGGGATAC	ATTAAAACTTTGATGCCTAC	CAPS	*Mfe*I
InDel6	ACAATCCCAGTTTATGTGAT	ATATTTGGTGTTTTCTGTTT	InDel	
HP2501	CTTTTCACAAAACTAACACAGG	TGACAATATAAGCATTTGTCGC	InDel	
HP707	TCCGATGTAACATCACGCAA	GTTGATCACCTTCAGACAGC	InDel	
D67	AGCTTTTATAGCACGTACCG	CCATACTCTACTTATGCTGCAA	InDel	
HP2509	ACCTCGACACTGGTTCACTC	GTGACTCATATACACCCTTACCTA	InDel	
HP445	GAGAACATCTGTACCAGCCT	CAAGTATCTATATGCCTGACAAC	InDel	

## Supplementary Materials

Supplementary materials can be found at https://www.mdpi.com/1422-0067/20/9/2204/s1.
