# Supplementary material for: Identification and Characterization of EI (Elongated Internode) Gene in Tomato (Solanum lycopersicum)
Source: Int J Mol Sci. 2019 May 5;20(9):2204. doi: 10.3390/ijms20092204 (PMC6540210; doi:10.3390/ijms20092204)
Supplement: Supplementary file 1 [file ijms-20-02204-s001.zip › Supplemental Materials/Supplemental table.docx]

**Table S1.** Primers used for qRT-PCR and sequencing.

| **Primer** | **Forward primer (5'-3')** | **Reverse primer (5'-3')** |
| --- | --- | --- |
| Actin^a^ | GGGATGGAGAAGTTTGGTGGTGG | CTTCGACCAAGGGATGGTGTAGC |
| EI^a^ | GGGAAGTTACCGTTGGGGAA | GAGCTTAGGGTTGTTGGGTGA |
| SlCPS^a^ | ATACCTAGAGCTAGCGAAATC | ACTGCCTAAATAGTACGTAACC |
| SlKS^a^ | TGATTTCTTTGATGTAGGAGGTTC | GCTTGCCACTTAGATGCTTTG |
| SlKO^a^ | CCACGAAGACACGCAGGTAG | ATCGTTCAGGCTTCCACTCTT |
| SlKAO^a^ | CTTTCAAATCCAACAATCCTG | TTAAAACCTTCCTGCAACCT |
| SlGA20ox1^a^ | CTCATTTCTAATGCTCATCGT | GTGAGATGATTCTTTCTTAGCG |
| SlGA20ox2^a^ | TTTCCATATTCTACCCTACAAG | TCATCGCATTACAATACTCTT |
| SlGA20ox3^a^ | AGCCAAATTATGCTAGTGTTAC | TTTTATGAGATTTGTGTCAACC |
| SlGA20ox4^a^ | GATGATAAATGGCACTCTATTC | TGACTTCCTTGTTCTTCTACAG |
| SlGA3ox1^a^ | GGCATTAGTAGTTAATATAGGTGA | AAATAAGCTACAGAAAGTCGATA |
| SlGA2ox1^a^ | GGCATGTAAGATATTAGAATTGA | TTAATCCGTAGTAGAGAATCAGA |
| SlGA2ox2^a^ | ATTAAGATCCAATAACACTTCG | TCTTGATTTCACACTATTTGC |
| SlGA2ox3^a^ | GACCCTTCTACTTTCAGCTC | AAATTGAATTGTCTTCTATCCA |
| SlGA2ox4^a^ | ATGGAAGGAAAAGACAGTTTA | CTTTTCTCAAATAGGACCAAC |
| SlGA2ox5^a^ | GATCACTTACCAATAATCAACAG | CGTCATGGTTTACGACTTTA |
| A1^b^ | GTGTTCTTCTTCAATCCTTTAC | CATTGTATCACGATGGGTAA |
| A2^b^ | AACAGATTGCGTTACCGTAT | AGGAAGTTAGGTAGGTAGGGT |
| A3^b^ | TATTTGTGAATCATCTTGGGC | TTCATCATCCCTGCTTATCTT |
| A4^b^ | CAGAAGATTGACAGAGCGAAAC | GTCCTTTGATCCGATGGTCTT |
| A5^b^ | CCGATTTAATAGGAATGAACT | TCGGCAGATCCTAAAGTAAAG |
| A6^b^ | GAGTAGTTGGCTGCCGATAAT | AACCCATCATGTGCAGATACA |
| A7^b^ | TCATAATATCTAACTACACCCAA | CTTGCTTCGTTTAGAATTAACAT |
| A8^b^ | ATCAAGATGAGATTGGAGGGT | GACTCAATATGGAGGAGGAAA |
| CDS^b^ | ACCAAAAGATAAGCAGGGAT | CATAGGATGGGCATAAGAAA |

^a^Primers for qRT-PCR ^b^Primers for sequencing
