# Supplementary figures and images for: Identification and Characterization of EI (Elongated Internode) Gene in Tomato (Solanum lycopersicum)
Source: Int J Mol Sci. 2019 May 5;20(9):2204. doi: 10.3390/ijms20092204 (PMC6540210; doi:10.3390/ijms20092204)

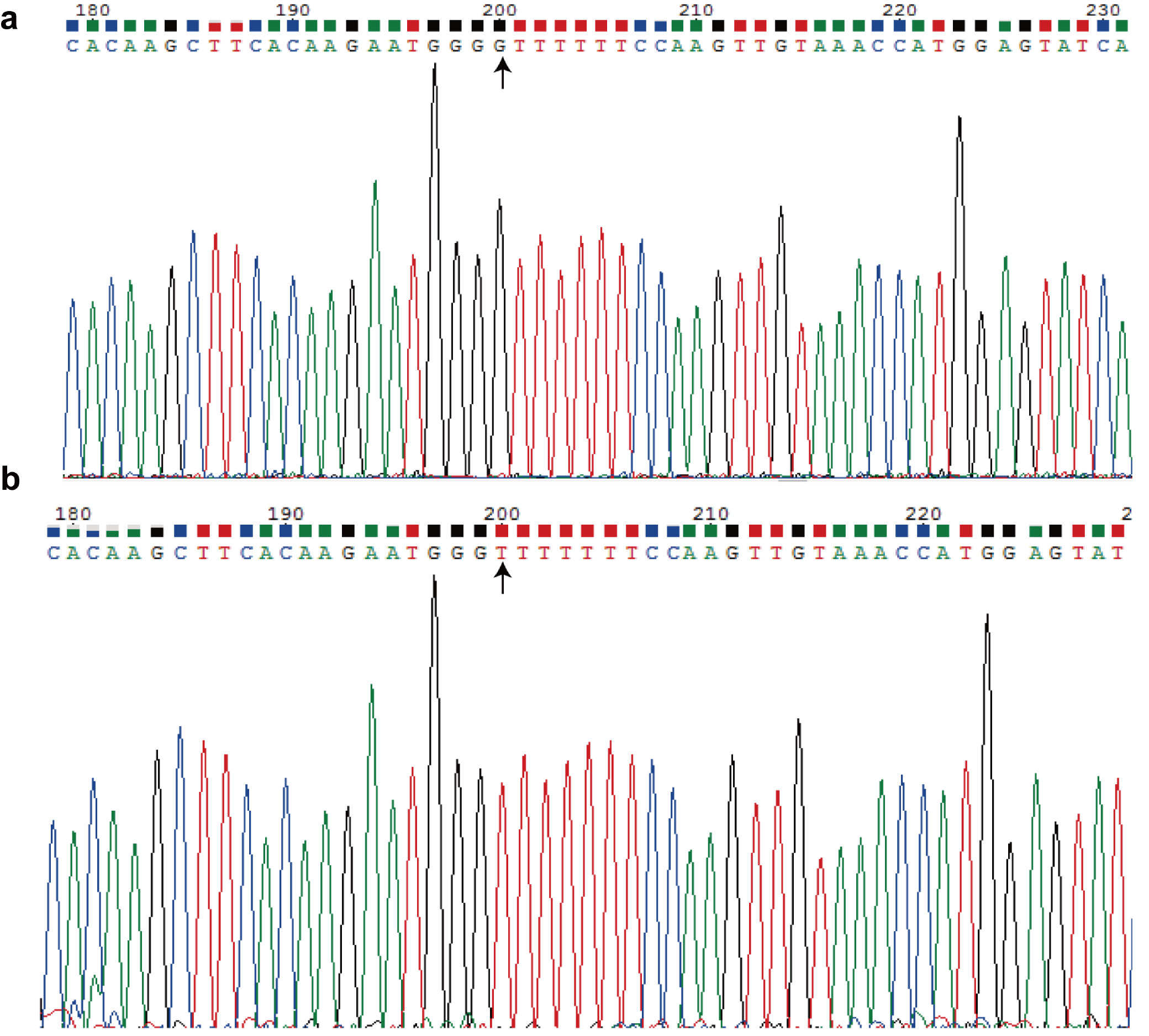

Supplement: Supplementary file 1 [file ijms-20-02204-s001.zip › Supplemental Materials/Figure.S1.tif]

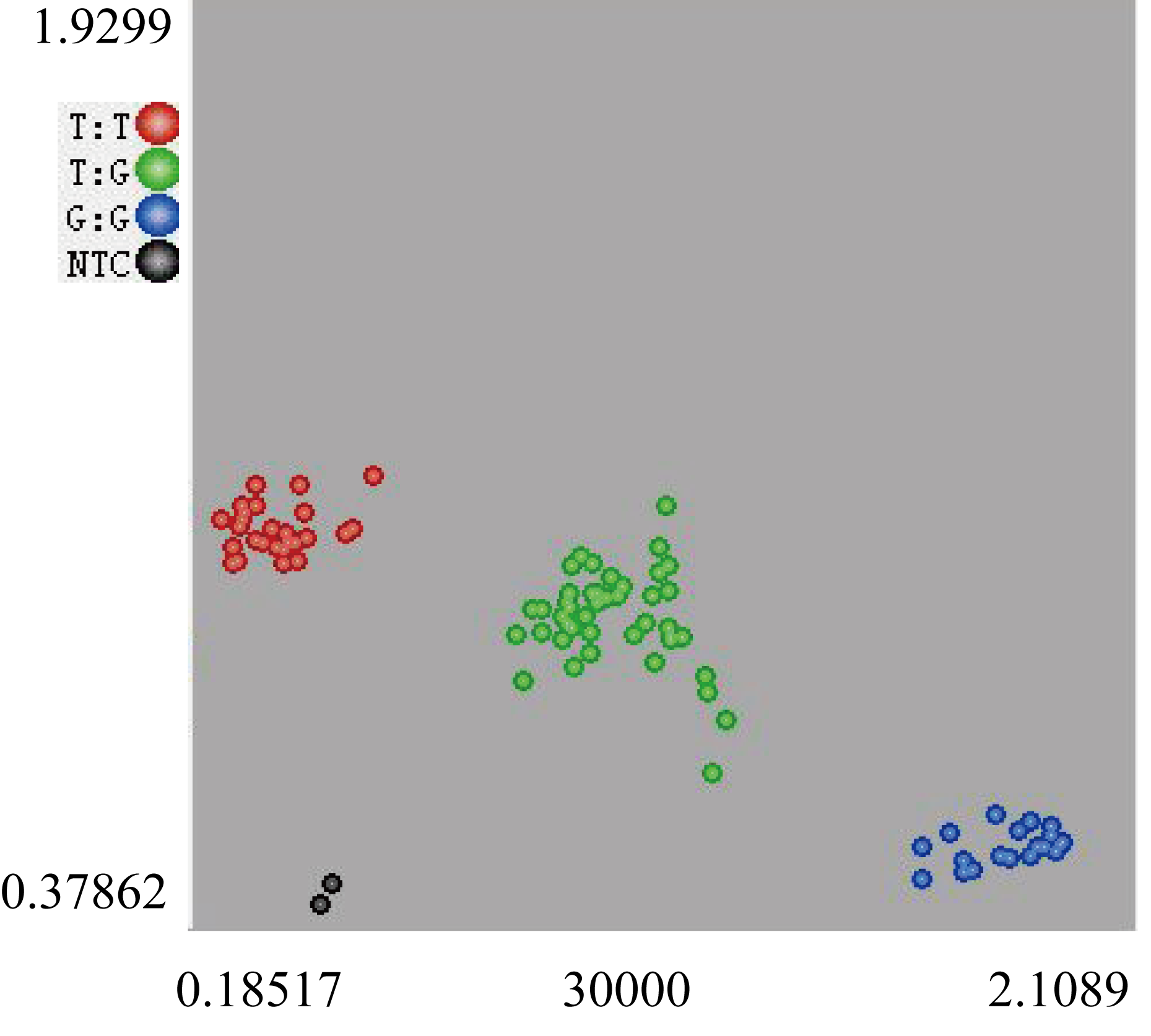

Supplement: Supplementary file 1 [file ijms-20-02204-s001.zip › Supplemental Materials/Figure.S2.tif]
